# Medical and public health professionals’ perceived facilitators and barriers of human papillomavirus (HPV) vaccination among African American adolescents in Shelby County, Tennessee

**DOI:** 10.1186/s12913-023-09415-6

**Published:** 2023-05-10

**Authors:** Seok Won Jin, Daniel Cruz Lattimore, Eric Harlin, Levonna Davis, Virginia Erholtz, Heather M. Brandt

**Affiliations:** 1 School of Social Work, The University of Memphis, 119 McCord Hall, Memphis, TN 38152 USA; 2 Department of Medical Humanities and Social Science, College of Medicine, Yonsei University, 50-1 Yonsei-ro, Seodaemun-gu, Seoul, 03722 Republic of Korea; 3 Institute of Media Arts , Yonsei University, 50 Yonsei-ro, Seodaemun-gu, Seoul, 03722 Republic of Korea; 4 The University of Memphis, 226 McCord Hall, Memphis, TN 38152 USA; 5 HPV Cancer Prevention Program, St. Jude Children’s Research Hospital, 262 Danny Thomas Place, Memphis, TN 38105 USA

**Keywords:** Adolescents, African Americans, Health Professional, Human papillomavirus vaccine, Qualitative research

## Abstract

Human papillomavirus (HPV) infects nearly 85% of sexually active Americans during their lifetime, causing most cervical and five other cancers. Routine HPV vaccination is recommended for adolescents to prevent HPV-attributable cancers, but HPV vaccination coverage remains low, especially in Tennessee. In 2021, 54.6% of the population in Shelby County, Tennessee was Black or African American, reporting higher rates of new cervical cancer cases than other counties in Tennessee. While medical and public health professionals (HPs) play a critical role in promoting vaccination coverage, little is known about the factors HPs perceive to influence HPV vaccination for this population. This study sought to explore HPs’ perceived facilitators and barriers of HPV vaccination among African American adolescents. Qualitative individual interviews with 26 HPs in Shelby County were conducted between October 2019 and February 2020. Interpretive content analysis of the interview data guided by the socio-ecological model revealed several important themes regarding the facilitators and barriers across the individual, interpersonal, and community levels. At the individual level, parental vaccine hesitancy emerged as a leading barrier to HPV vaccination, while appropriate education facilitated the vaccination. At the interpersonal level, a lack of strong provider recommendations impeded HPV vaccination, whereas improved communication skills with patients facilitated the vaccination. Finally, the community-level barriers included a lack of education and social/religious norms; the community-level facilitators included community outreach efforts. HPs should consider development of comprehensive community-based approaches that leverage the facilitators and barriers at multiple levels to increase HPV vaccination among African American adolescents in this region.

**Supplementary Information:**

The online version contains supplementary material available at 10.1186/s12913-023-09415-6.

## Introduction

Human papillomavirus (HPV) is the most common sexually transmitted infection in the United States (U.S.) [1]. HPV infects about 14 million American teens and adults annually, and nearly 85% of sexually active Americans through intimate skin-to-skin contact during their lifetime [2]. HPV can cause genital warts, most cervical cancers and precancers of the cervix, and the majority of cancers of the oropharynx, vagina, vulva, penis, and anus for both men and women [3]. Every year in the U.S., more than 37,000 men and women are diagnosed with cancers caused by HPV [4], which poses a substantial financial burden (approximately $775 million in direct medical costs) on the U.S. healthcare system [5].

To prevent HPV-attributable cancers, the Advisory Committee on Immunization Practices (ACIP) recommends routine HPV vaccination on the following schedule: two doses (6–12 months apart) for adolescents aged 11–12 years, starting at age 9, and three doses (given over 6 months) for individuals who receive their first dose at age 15 through 26 years [6]. The ACIP also recommends HPV vaccination for some adults aged 27 through 45 years with shared clinical decision-making [6].

Despite the availability of a safe, effective, durable vaccine against HPV, HPV vaccination rates remain lower than optimal in the U.S., falling far below the 80% benchmark of the Healthy People 2030 national goal [7]. A recent National Immunization Survey-Teen (NIS-Teen) found that in 2021, 61.7% of adolescents—59.8% of males and 63.8% of females—aged 13 through 17 years were up to date with the HPV vaccination series [8]. Further, sub-analyses of the NIS-Teen data by region revealed that the state of Tennessee (TN) had only 56.5% of the target age group up to date with the vaccination series, becoming the 10th lowest state in the U.S. [9]. According to the Tennessee Immunization Information System (TennIIS), 31.5% of adolescents aged 11 to 17 in Shelby County were up to date with the vaccination series in 2022, while 29.6% of African American adolescents aged 11 to 17 in this region were up to date with the series in the same year [10]. In 2021, 54.6% of the population in Shelby County, Tennessee was Black or African American, reporting higher rates of new cervical cancer cases than other counties in Tennessee [11].

A systematic review and meta-analysis study indicated that African Americans had reported lower rates of completion of the HPV vaccination series than non-Hispanic whites [12], while African Americans had experienced high rates of genital and oral HPV prevalence, vaginal and anal cancer incidence, and cervical cancer mortality [13, 14]. According to existing literature, the underuse of HPV vaccines among African Americans is associated with multiple factors. For sociodemographic factors, African Americans who identified as male, lived in rural areas with few healthcare providers, or had no health insurance, were likely to report low rates of HPV vaccination [15]. Moreover, low HPV vaccination coverage in African Americans was connected to their psychosocial characteristics, including a lack of knowledge regarding HPV, negative attitudes and beliefs about HPV vaccination, and parental concerns about the vaccine safety or potential side effects [16]. Also, African Americans’ medical mistrust of healthcare providers and the healthcare system contributed to their underutilization of healthcare services, including HPV vaccination [17], while provider recommendations for HPV vaccination were a key factor of parental intention on vaccinating their child(ren) against HPV [18]. Finally, limited provider recommendations and the costs related to vaccination prevented African Americans from receiving HPV vaccinations [19].

As shown in the literature, research has highlighted the potential of medical and public health professionals, including healthcare providers or community health workers to influence African American parents’ decision-making for adolescent HPV vaccination at multi-faceted venues. However, little is known regarding which factors medical and public health professionals (hereafter, health professionals) perceive to influence adolescent HPV vaccination, especially in the African American community. This is essential to designing interventions for HPV vaccine promotion with this community. Thus, this study sought to explore health professionals’ perceived facilitators and barriers to HPV vaccination for African American adolescents in Shelby County, TN.

## Methods

### Study design

A qualitative design of face-to-face individual interviews was employed to explore health professionals’ perceived facilitators and barriers regarding HPV vaccination for African American adolescents. For this study, a research team consisting of three African American doctoral students (a female and two males) and a white female undergraduate student at a public university in Shelby County, TN worked with faculty to inform the study design and execution. All authors, including students, successfully completed a human subject research ethics training provided by the Collaborative Institutional Training Initiative Program and received training on administrating a consent form, conducting individual interviews, collecting and managing qualitative data, and analyzing data provided by the first author. The study was approved by the first author’s university institutional review board (FWA00006815, IRB ID: #PRO-FY2020-70).

### Sampling and settings

Purposive sampling was performed to recruit health professionals from diverse community settings including local clinics, hospitals, the county health department, health organizations, and universities. Inclusion criteria for this study required participants be aged 18 years or older and self-identified as a health professional in Shelby County, TN (e.g., primary care doctors/nurses, public health workers, community health educators, or university/college faculty) working mostly with African Americans. For recruitment, the research team held a series of brainstorming sessions to develop a list of health-related organizations and institutions in Shelby County, TN and collected contact information (i.e., email addresses and office phone numbers) from public websites or personal referrals. A total of 79 potential participants from this list were contacted via email or phone to obtain their permission to conduct an individual interview and schedule an interview with those who agreed to participate. The concepts of saturation and sufficiency were used to determine sample size.

### Data collection

A semi-structured interview guide of open-ended questions was developed by the research team to collect qualitative data regarding facilitators and barriers to HPV vaccination among African American adolescents in Shelby County, TN. In this study, the term ‘HPV vaccination’ referred to both the initiation and completion of the vaccine [20–22]. For data collection, three trained African American doctoral students conducted individual interviews with respondents. Prior to interviews, all participants completed informed consent forms. Interviews used a funnel approach to ask a broad question followed by more specific questions to probe respondents’ answers. The data collection stopped at 26 interviews having reached saturation of key terms when no new data or information was being obtained across all the health professional groups [23]. All interviews occurred between October 2019 and February 2020 at participants’ preferred locations to maximize a comfortable, natural interview environment. Each interview was completed in an average of 35 min. All interviews were audio-recorded with participants’ permission. Brief demographic information was collected from all respondents, including their professional titles—i.e., (7) medical doctors, (10) nurses/nurse practitioners, (3) university faculty, and (6) public health workers. Table 1 presents a summary of the respondent demographics. Respondents received a $30 gift card as an honorarium.

**Table 1:** Demographic characteristics of respondents (*N* = 26)

Characteristics	Category	Number	%
Age group	Under age 45	6	23.1
	45–54	5	19.2
	55–64	12	46.2
	65 or older	3	11.5
Race	Non-Hispanic Black	8	30.8
	Non-Hispanic white	8	30.8
	Unknown/Refused to answer	10	38.5
Gender	Male	3	11.5
	Female	23	88.5
Education	College degree	8	30.8
	Graduate degree or above	18	69.2
Organization/Institution	Health clinic	6	23.1
	Hospital	8	30.8
	Health organization	2	7.7
	University	3	11.5
	County health department	7	26.9
Title	Medical doctor	7	26.9
	Nurse/Nurse practitioner	10	38.5
	University faculty	3	11.5
	Public health worker	6	23.1

### Data analysis

**Fig. 1:**
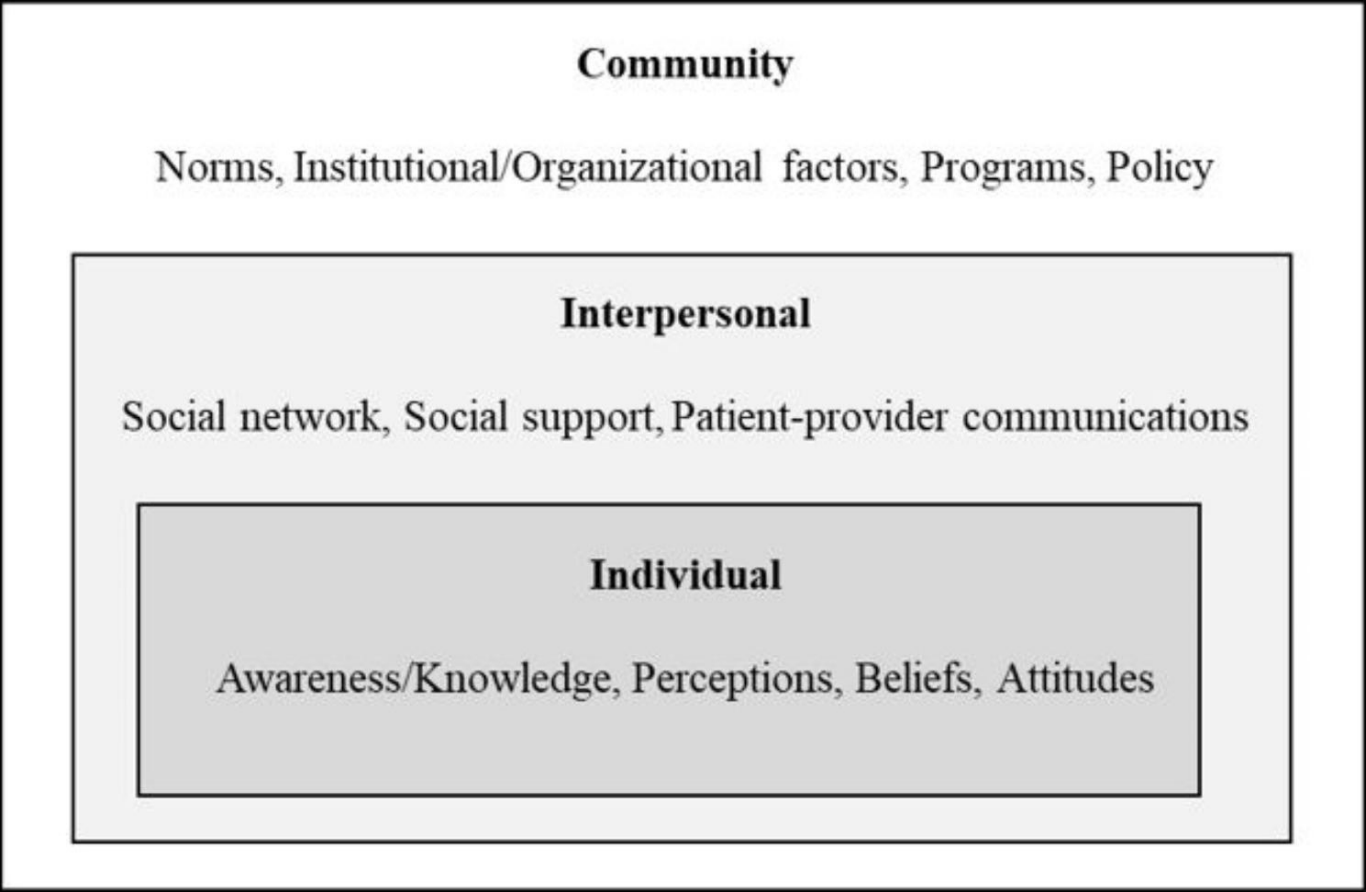
Socio-ecological model as an analytic framework

All interview data were transcribed verbatim and analyzed in a three-stage process using inductive coding and qualitative content analysis building on the socio-ecological model (SEM) as a conceptual framework [21, 24, 25]. The SEM posits that individual health outcomes result from reciprocal interactions between a person and the person’s environment at multiple levels [26]. The SEM provides organization of codes for assessing multi-level and bidirectional influences, which allows for understanding different levels of influence and potential bidirectional interaction across and between levels [27, 28]. This is useful for understanding the state of HPV vaccination among African Americans from the perspective of healthcare providers [24]. In this study, the SEM included the three levels of influence—individual (knowledge, perceptions, beliefs, and attitudes), interpersonal (social network, social support, and patient-provider communications), and community (norms, institutional/organizational factors, programs, and policy) levels—to describe health professionals’ perceptions toward the barriers and facilitators of HPV vaccination among African American adolescents (See Fig. 1).

In the first stage, the first author and the three interviewers read and reviewed assigned transcripts multiple times to fully understand the responses before coding. As a coder, each interviewer independently conducted first- and second-level coding to generate a joint codebook which summarized all interview concepts and included corresponding direct quotes. Next, all coders reviewed the codes as a group and resolved discrepancies through discussion and consensus. In the second stage, the coders revisited the transcripts and codebook to conduct third-level coding to finalize and categorize the codes into either barriers or facilitators. In the final and third stage, the coders repeated the first-stage process again, categorizing all codes according to the barriers and facilitators at the three SEM-based levels, respectively, and identified emerging themes by building relationships among the codes and categories through iteratively comparing the results.

### Findings

#### Facilitators to HPV vaccination

As shown in Table 2, analyses of the interview data revealed several themes associated with facilitators that could reduce barriers to HPV vaccination and increase vaccination among African American adolescents across the individual, interpersonal, and community levels.

**Table 2:** A summary of themes by barriers and facilitators at the SEM-based levels

Level	Barrier	Facilitator
Individual	• Parental vaccine hesitancy - Medical mistrust ♣ Concerns about safety - Misinformation ♣ No perceived need ♣ A lack of awareness	• HPV vaccination education utilizing appropriate content
Interpersonal	• A lack of provider recommendations • A lack of recommendation strength	• Provision of provider recommendations • Improved communication skills with patients
Community	• A lack of public education • Social and religious norms • No policy requirement in schools • Issues in the healthcare system	• Enhanced accessibility in communities • Community outreach efforts • Introduction of a vaccine requirement policy

#### Individual-level facilitators

HPV vaccination education utilizing appropriate content emerged as an individual-level facilitator. A respondent stated that the educational content should cover basic information about HPV, including HPV-attributable diseases, vaccination schedules, and vaccine safety. Another respondent recommended creating simple, accessible educational content including statistical information on HPV prevalence, mortality, and vaccination benefits.

#### Interpersonal-level facilitators

The interpersonal-level facilitators included provision of recommendations and improved communication skills with patients.

**Provision of provider recommendations.** Multiple respondents stated that provider recommendations could facilitate HPV vaccination among African American adolescents. In regard to provider recommendation, a respondent said, “Usually favorable. I mean if I make the recommendation, the patient will usually go ahead and get the shot.” Another respondent said, “You make a recommendation. It’s like, ‘Yes, doctor! We’ll do it.’”.

**Improved communication skills with patients.** Several respondents noted that improved communication skills among healthcare providers could promote HPV vaccination in African American adolescents. For example, a respondent said, “So, I think it’s getting the children into their provider, their provider being knowledgeable about the vaccine, how to talk about it, how to recommend it, right.” Another respondent indicated that a clear message highlighting the effectiveness of HPV vaccines on cancer prevention was important to promote HPV vaccination for African American adolescents.

#### Community-level facilitators

Respondents reported community-level facilitators including: enhanced accessibility to HPV vaccination, increased community outreach efforts within the African American community, and introduction of a vaccine requirement policy.

**Enhanced accessibility in communities**. Respondents viewed enhanced accessibility to HPV vaccines in community settings as a facilitator of HPV vaccination for African American adolescents. A respondent commented that individuals should be able to easily access providers to receive HPV vaccines in their neighborhoods, saying, “You know, people can’t be trying to buy [take] a bus an hour across city to see the doctor.” Another respondent recommended making HPV vaccines accessible at community pharmacy stores. One respondent noted that providing free HPV vaccines could help increase vaccine coverage, saying, “Because they receive these vaccines free from the federal government for children who would otherwise not receive vaccines because their parents can’t afford the vaccines. Or they’re uninsured.”

**Community outreach efforts**. Several respondents stated that community-wide outreach efforts could increase HPV vaccination among African American adolescents. For example, a respondent commented that various community-based approaches to educating residents on HPV vaccination could facilitate the vaccination in the African American community. The respondent said, “Take a section of the city and go out and hand out. Door to door or maybe have a community event and have a table set up just where you would have somebody knowledgeable. And develop some kind of a flyer or something.” Additionally, multiple respondents indicated a need for targeted social marketing and campaigns for HPV vaccination. For example, one respondent said, “I guess the main strategy is, you know, these kind of big community-wide efforts, whether that’s through, you know, mass media, social media, community-based interventions, and then also healthcare-based intervention, right.” Also, respondents commented that a mobile immunization clinic or commercial-based community program could improve HPV vaccination rates in the community.

**Introduction of a vaccine requirement policy**. Multiple respondents mentioned that HPV vaccination for African American adolescents could improve with the implementation of policy to require HPV vaccination for school attendance. A respondent said, “Schools have to have buy-ins. Governments have to have buy-ins and local governments have to have buy-ins because some parents aren’t going to like that.” Another respondent said, “Some states have gone to requiring it for school entry. I don’t think … that’s likely to happen anytime soon in Tennessee, but that is a nationwide that’s an avenue that some states have taken.”

### Barriers to HPV vaccination

As shown in Table 2, analyses of the interview data revealed several themes regarding health professionals’ perceived barriers to HPV vaccination for African American adolescents at the individual, interpersonal, and community levels.

#### Individual-level barriers

Respondents reported individual-level barriers including vaccine hesitancy, medical mistrust, vaccine safety concerns, misinformation, no perceived need, and a lack of awareness. In sub-analyses, vaccine hesitancy emerged as a leading barrier at the individual level, while medical mistrust was connected to vaccine safety concerns, and misinformation was connected to no perceived need and a lack of awareness, respectively.

**Vaccine hesitancy.** Respondents indicated that some African American parents’ hesitancy for HPV vaccines was a primary barrier to adolescent HPV vaccination. One respondent stated that when HPV vaccines were recommended for children, African American parents often expressed a need for time, saying, “I need to talk to my husband/wife” or “I need to do some research.” The respondent also added that these requests usually led to “just a delay” of vaccination for their children. Another respondent expressed feeling frustrated, stating, “Sometimes, I just feel like I failed because it’s just so hard to get across to that population.”

**Medical mistrust.** Several respondents stated that medical mistrust prevented African American parents from vaccinating their children against HPV. Specifically, the analysis showed that medical mistrust interrupted the influence of provider recommendations on vaccine-related decision-making among African American parents. One respondent said, “Some patients feel that … whatever we recommend is going to hurt them.” Also, the analysis revealed that this medical mistrust stemmed from a belief that African Americans could be used as test subjects in unknown medical experiments without their consent and/or without being fully informed. An African American respondent said, “A lot of Blacks feel that … you’re their guinea pig, you’re that white mouse, that white rat being tested. So, they’re shooting something in you, being a black person to see the reaction.”

**Concerns about safety.** Multiple respondents noted that African American parents were concerned about the safety of HPV vaccines which was linked to parents’ anxiety and/or fear pertaining to the administration of the vaccine to their children. For example, one respondent said, “I know … concerns that it’s [the vaccine’s] not safe.” An African American respondent also said, “To be honest, we as Black people … we are afraid of shots.” The respondent indicated parent’s concerns, saying “So I can say again, ‘fear of the unknown.’ … [Parents think] they [doctors]’re putting this infection, these viruses into our children. That’s how they’re so sick.”

**Misinformation.** Respondents viewed misinformation about HPV vaccines as a potential barrier to HPV vaccination for African American adolescents. This misinformation included vaccine-related side effects among adolescents, including autism and cancer. One respondent said, “… over the years of interacting with our patient population, we’ve seen increasingly more individuals who have sort of being indoctrinated with misinformation about safety issues associated with HPV.” Some respondents reported, “Children became autistic after they got the vaccine and I’ve seen a lot of Facebook post about that,” “We’ll have folks who come and say, well, this [HPV vaccine] caused …this cancer in them or other things,” and “Patients that have looked up information online for themselves, and then for that reason they did not get the HPV vaccination.” Also, respondents indicated that misinformation spreads quickly from person-to-person through social media platforms, including Facebook. One respondent said, “People just pass it on and negativity travels really fast.” Another respondent added, “They may have misinformation they might have got from the Internet or talking to neighbors.” Multiple respondents commented that many of their patients, including African Americans had obtained misinformation about the HPV vaccine from online sources which had a greater influence on their vaccine-related decision-making than did the information provided by their healthcare providers. For example, one respondent said, “The Internet has played a role in hesitancy with the HPV [vaccine].” Another respondent added, “So they are kind of believing more of what’s online than what they’re being taught from their physicians.”

**No perceived need.** Several respondents noted that many African American parents perceived no need for HPV vaccination for their children because they believed that their child was not sexually active yet. A respondent stated that African American parents often linked HPV to sexual activity, saying “They had the impression that it really was not applicable to their child because their child was not sexually active.” Another respondent said, “My child has not, [is] not going to have sex until marriage and they’re not going to marry anyone who’s ever had sex before. So, we don’t have to worry about that.”

**A lack of awareness.** Respondents indicated that a lack of awareness of HPV vaccination among African American parents was a barrier to the vaccination for their children. For example, a respondent said, “I mean, because a lot of people at first had not even heard of HPV. They were like, is that HIV? … What is that?” Another respondent said, “Immunizations [are] just not really on their radar.”

#### Interpersonal-level barriers

The interpersonal-level barriers respondents reported included a lack of provider recommendations and a lack of recommendation strength.

**A lack of provider recommendations.** A lack of provider recommendations emerged as one of the interpersonal-level barriers. For example, a respondent said, “We noted that parents were not being prompted by their local providers to get HPV immunization.” Another respondent captured their experience with patients, saying, “Did your pediatrician mention it to you? No. Has your OBGYN [Obstetrics and Gynecology] from somewhere else mentioned it to you? No. Your family practitioner? No. That’s most of the patients’ answers.” A respondent attributed the lack of recommendations to providers’ perceptions toward HPV vaccination, saying “I think it [HPV vaccination] at times isn’t a priority for providers.”

**A lack of recommendation strength.** Respondents commented that provider recommendations lacked appropriate strength, which might hinder HPV vaccination among African American adolescents. For example, a respondent said, “When we offer the [HPV] vaccine, it’s offered as a choice.” Another respondent said, “It [HPV vaccine] is not really being promoted by their primary care providers as something that’s necessary.” Additionally, a respondent said, “They [Healthcare providers] don’t routinely promote HPV vaccines. And even when they come in for that 11-to-12-year visit, when they’re getting meningococcal and TDaP, it’s like, ‘OK, you have to have these.’ And they either say, ‘You don’t have to have the HPV [vaccine],’ or they say, ‘Do you want the HPV [vaccine]?’”.

#### Community-level barriers

Respondents reported community-level barriers including a lack of vaccine-related public education, social and religious norms, no policy requirement in schools, and issues in the healthcare system.

**A lack of public education.** Respondents identified a lack of education about HPV and the vaccine in the African American community as a barrier to adolescent HPV vaccination for African American parents. One respondent said, “The public didn’t really know a lot about it [HPV].” Another respondent said, “Well, the number one thing for me is education.”

**Social and religious norms.** Respondents stated that social and religious norms interfered with receiving HPV vaccination in the African American community. A respondent stated that African American parents were worried about the possible stigmatization associated with HPV and believed that giving their child the HPV vaccine was akin to giving them permission to have sex. Furthermore, multiple respondents indicated HPV was a taboo topic in their religious communities. For example, a respondent said, “I think a big barrier is that we don’t want to [talk about HPV] because for religious reasons.” Another respondent said, “People don’t talk about that stuff [HPV] because it’s a place that claims to be religious, so they don’t talk about it. So, their religious affiliation can put them at risk, for things like this, HPV.”

**No policy requirement in schools.** Respondents identified the absence of policies requiring the HPV vaccination in schools as a barrier. Multiple respondents noted that many African American parents did not want to vaccinate their children against HPV if doing so was not compulsory. For example, a respondent said, “They’ll come with their school forms showing what’s required as far as the immunizations to get into their colleges and universities and they’ll say, ‘Oh, this one isn’t required. So, we don’t want that one.’ And that’s 9 times out of 10. That’s the HPV.” Additionally, a respondent stated that no requirement of HPV vaccination in schools might imply to parents that the HPV vaccination was not important because they believed that schools prioritize and/or mandate public health.

**Issues in the healthcare system.** Respondents expressed several issues in the current healthcare system regarding HPV vaccination. A respondent reflected on the limitations healthcare providers faced when trying to track the vaccination history of children, mainly because the HPV vaccine was inaccurately included in the electronic medical record (EMR). The respondent stated, “There is a Tennessee registry where we—for at least the last five years, maybe three years—have been dumping all our information when we give vaccines. It doesn’t communicate yet with our EMR.” Furthermore, multiple respondents pointed to another barrier: insufficient time for communication with patients. For example, a respondent said, “The pediatricians, they’re so busy with the things that they gotta get done.” Another respondent stated, “And part of that, too, is time because of the way the schedules are set up in the offices. Your schedule is stacked and all you are allotted is fifteen minutes per patient.” Moreover, a respondent identified the cost of the HPV vaccination as a barrier. Finally, several respondents noted that a lack of transportation hindered many African American adolescents from accessing HPV vaccinations in the community. For example, a respondent said, “Some of the barriers, there are people can’t just they can’t get to the doctor’s office. They don’t have transportation.” Another respondent said, “Sometimes the parents are just, ‘I have seven kids, I don’t have time for that. I’m the only one provider providing I don’t have anyone take care of my little ones. I don’t have any transportation.’”.

## Discussion

This research team explored medical and public health professionals’ perceptions toward HPV vaccination among African American adolescents in Shelby County, TN by applying the SEM as a conceptual framework. Several themes emerged regarding facilitators and barriers pertaining to HPV vaccination across the individual, interpersonal, and community levels. The findings were consistent with the existing literature. The findings also contribute to the addition of important knowledge in the current body of research on HPV vaccine practices in the African American community, especially in the Shelby County, TN, which has reported high rates of new cervical cancer cases and suboptimal rates of HPV vaccination. Additionally, the findings provide implications for comprehensive community-based practices and future research to increase HPV vaccination coverage in this region.

At the individual level, the research team found that parental vaccine hesitancy was a leading barrier to HPV vaccination among African American adolescents. Prior studies also identify vaccine hesitancy as a top barrier to initiation and completion of the HPV vaccination series [29–31]. Also, the research team discovered that HPV vaccination education utilizing appropriate content was an individual-level facilitator of the vaccination. Health communication experts point to the importance of using targeted messages and educational materials in vaccine campaigns and education programs to increase vaccination coverage [32–34]. These findings suggest that medical and public health professionals need to develop HPV vaccination content targeting African American parents to influence vaccine-related decision making for their children. Particularly, the present study revealed how vaccine hesitancy in African American parents is connected with other individual-level barriers, including medical mistrust (associated with vaccine safety concerns) and misinformation (coupled with parental perception that the vaccine is not necessary until their child is sexually active and a lack of vaccine awareness). The Strategic Advisory Group of Experts on Immunization Working Group on Vaccine Hesitancy indicates that vaccine hesitancy is complex, context-specific, and varies by vaccine, classifying these barriers as determinants of vaccine hesitancy [35]. Therefore, medical and public health professionals should ensure that the educational content designed to target HPV vaccine hesitancy effectively addresses these barriers to reduce parental levels of HPV vaccine hesitancy. For example, because medical mistrust in African Americans is deeply rooted in historical events and experiences, such as the U.S. Public Health Service Syphilis Study at Tuskegee and additional historical and recent examples [36], HPV vaccine hesitancy among African American parents should be understood within this historical and present-day context. Additionally, previous studies indicate that people with medical mistrust tend to seek health-related information outside of the traditional healthcare system, and instead, rely heavily on misinformation online, which may aggravate the degree of vaccine-related safety concerns, misperceptions, and/or knowledge, leading to increased vaccine hesitancy [37–39]. Future research should investigate the mechanisms underlying the associations among these barriers and their paths to the HPV vaccine hesitancy in African American parents.

At the interpersonal level, the research team found that the lack of provider recommendations and strength of the recommendation prevented African American adolescents from receiving HPV vaccination, whereas both provision of the provider recommendations and improved communication skills with patients facilitated their HPV vaccination. Substantial research demonstrates that provider recommendations are one of the strongest predictors of the vaccination for adolescents [40–44]. Multiple studies show that strong provider recommendation, in which presumptive language is used, is effective in influencing parents’ intentions and decision making on HPV vaccination of their children [45–47]. Additionally, prior research indicates that healthcare providers are the most trusted source of vaccine information among parents and adolescents [48, 49]. Taken together, healthcare providers in clinical settings should continue to recommend that the HPV vaccine be administered to adolescents during office visits, otherwise parents may assume the vaccine is not needed. Given the individual-level barriers, however, medical mistrust in vaccine-hesitant African American parents may undermine the effect of the recommendations from healthcare providers on their intention or decision to vaccinate their child. Culturally competent communication between providers and vaccine-hesitant African American parents which focuses on the effectiveness of the HPV vaccine in cancer prevention, are warranted [50]. Practitioners and researchers should both develop strategies for effectively communicating with vaccine-hesitant African American parents and investigate the effectiveness of the communications in reducing parental hesitancy for HPV vaccines and improving parental beliefs and intentions about the vaccination for their children.

At the community level, the research team found that the barriers to HPV vaccination included a lack of education in the community, social and religious norms, no requirement for school enrollment, and issues in the healthcare system, while the facilitators included enhanced accessibility in communities, community outreach programs, and introduction of a vaccine requirement policy. These findings illuminate several implications for community-based approaches to increase HPV vaccination in African American adolescents. First, HPV vaccination education should address social and religious norms including stigma toward HPV infection and relevant cancers and the taboo of discussing HPV-related topics as a way to normalize HPV vaccination for cancer prevention [51–53]. Collaborative partnerships with religious leaders are essential to increase HPV vaccination in the African American community. Second, schools have the potential to educate students on HPV and the benefits of vaccination. School-based vaccine education should consider a gender-neutral approach, focusing on the causes of HPV infection (e.g., intimate skin-to-skin contact) and HPV-associated cancers for both men and women (e.g., annually in the U.S., about 37,000 cases of HPV cancers, 200,000 pre-cancers of the cervix, 500,000 cases of genital warts, 11,800 cases of cancer in the back of the throat in men, which surpasses 2,200 cases of cancer in the back of the throat in women; HPV vaccination prevents 90% of these pre-cancers and cancers and almost all cases of genital warts in the U.S.) [54–56]. Additionally, it is notable to explore the role of school-entry mandates on HPV vaccination as a state-level policy. Some states currently require HPV vaccination for school entry and report higher rates of HPV vaccination among adolescents compared to those without this policy [57–59]. As previously mentioned, African American parents may view schools as institutions that care for students’ health, which may cause already concerned parents to misinterpret a lack of vaccine policy as the HPV vaccine being unnecessary for their child. Finally, to enhance accessibility to HPV vaccination in the African American community, development of community outreach programs and interventions is needed. These programs and interventions should provide information about HPV vaccine access points in Shelby County, TN, including multiple pediatric practices, federally qualified health centers (FQHCs), the health department, clinics, pharmacies, and mobile onsite clinics (i.e., ShotRx901) [60]. It is worthwhile for future research to map these access points to facilitate parental navigation and increase access to HPV vaccination within the African American community. Because the current system does not guarantee sufficient time for communicating with patients in clinical settings, local pharmacies may contribute to closing the existing gap in vaccine communications between physicians and patients [61]. Pharmacists should also allow for dialogue with patients about HPV and the vaccination and offer recommendations [62–64]. These collaborative, community-wide efforts for HPV vaccination may reduce the barriers to HPV vaccination and increase HPV vaccination coverage in the African American community.

Most of the findings in this study are supported by prior studies among African Americans. A qualitative study by Fields et al. demonstrated that an explicit vaccine recommendation from a healthcare provider functioned as a moderator of HPV vaccination among African American women [65]. In addition to the vaccine recommendation, a literature review by Galbraith et al. revealed that medical mistrust, perceived lack of need for the vaccination, concerns about vaccine safety, and religious beliefs were associated with vaccination outcomes among African American women [66]. Particularly, Galbraith et al.’s study indicated healthcare provider’s vaccine hesitancy as a barrier to the vaccination among African American women [66], while the present study indicated a lack of the strength of a healthcare provider recommendation as a barrier to the vaccination. Moreover, the findings of this study expand the body of literature regarding HPV vaccination among African Americans by addressing community-based vaccination efforts. This study highlights the importance of improving public education, school entry policies, accessibility, and outreach programs for HPV vaccination in the African American community. Public health workers and policy makers should consider these findings when developing strategies for HPV vaccination promotion among African Americans.

## Study limitations

This study included some limitations that might influence the interpretations of the findings. Given the nature of qualitative research focused on a small number (26) of respondents from the target population (i.e., medical and public health professionals), these findings cannot be generalized to all African American adolescents in the U.S., and instead, should be carefully interpreted within the context of the study participants and location. Comparison studies with a detailed description may enhance the transferability of the findings [67]. Also, this study employed an individual interview approach to collect qualitative data from diverse medical and public health professionals in the community, so these findings might be subject to social desirability bias; this study might not fully capture respondents’ unconscious perceptions, beliefs, and attitudes toward HPV vaccine practices in the African American community [68]. Future studies using quantitative methods with a larger sample size should be considered to expand understanding of the facilitators and barriers of HPV vaccination to corroborate these findings.

## Conclusion

This study captured the perceptions of medical and public health professionals regarding HPV vaccination among African American adolescents in Shelby County, TN. Respondents provided insight into the facilitators and barriers to the vaccination of this population against HPV. The findings of this study offer a better understanding of what is needed to develop comprehensive, community-based approaches that leverage the facilitators and barriers at multiple levels to increase HPV vaccination among African American adolescents in this region.

## Electronic supplementary material

Below is the link to the electronic supplementary material.

Supplementary Material 1

## Data Availability

The data that support the findings of this study are available from the corresponding author upon request.
