# Supplementary material for: Medical and public health professionals’ perceived facilitators and barriers of human papillomavirus (HPV) vaccination among African American adolescents in Shelby County, Tennessee
Source: BMC Health Serv Res. 2023 May 10;23:469. doi: 10.1186/s12913-023-09415-6 (PMC10173571; doi:10.1186/s12913-023-09415-6)
Supplement: Supplementary file 1 — Supplementary Material 1 [file 12913_2023_9415_MOESM1_ESM.pdf]

INDIVIDUAL INTERVIEW PROTOCOAL

Topic 1	**Communication with Patients for HPV and HPV Vaccinations**<br>a. PROBE: Have you ever talked about HPV or HPV vaccinations with your African American patients?<br>b. PROBE: Have you ever recommended HPV vaccination to your patient?<br>c. PROBE: If so, what were the responses from the patents to your recommendation?
Topic 2	**Barriers and Facilitators to HPV Vaccination**<br>Now, we'd like to listen to your thoughts of the barriers and facilitators to the HPV vaccination among African American adolescents.<br>2. What do you think prevents adolescents from receiving HPV vaccines?<br>a. PROBE: tell me what are barriers to participating in HPV vaccination among adolescents?<br>b. PROBE: What concerns did your patients have about HPV vaccinations for their children?<br>3. What do you think helps adolescents receive HPV vaccines?<br>a. PROBE: What would be the most helpful for adolescents to come to participate in the HPV vaccination?<br>b. PROBE: What information should be provided to parents in order to help their children receive HPV vaccinations?<br>c. PROBE: What support do you think parents need to get their children receive HPV vaccinations?
Topic 3	**Strategies for HPV Vaccination Promotion**<br>Finally, we'd like to listen to your thoughts of strategies for promoting HPV vaccination among African American adolescents.<br>4. Tell me about your thoughts of strategies for promoting HPV vaccination among adolescents.<br>a. PROBE: What strategies should be developed to promote HPV vaccination among adolescents?<br>b. PROBE: Would you recommend any technology that helps adolescents receive HPV vaccines?<br>c. PROBE: What policies should be changed to promote HPV vaccination among adolescents?
Other	Does anyone have any final thoughts about the HPV vaccination for African American adolescents that you haven't gotten to share yet?
